# Highly Flexible Self-Assembled V_2_O_5_ Cathodes Enabled by Conducting Diblock Copolymers

**DOI:** 10.1038/srep14166

**Published:** 2015-09-22

**Authors:** Hyosung An, Jared Mike, Kendall A. Smith, Lisa Swank, Yen-Hao Lin, Stacy L. Pesek, Rafael Verduzco, Jodie L. Lutkenhaus

**Affiliations:** 1 Artie McFerrin Department of Chemical Engineering, Texas A&M University, College Station, TX; 2 Department of Chemical and Biomolecular Engineering, Rice University, Houston, TX; 3 Department of Materials Science and NanoEngineering, Rice University, Houston, TX

## Abstract

Mechanically robust battery electrodes are desired for applications in wearable devices, flexible displays, and structural energy and power. In this regard, the challenge is to balance mechanical and electrochemical properties in materials that are inherently brittle. Here, we demonstrate a unique water-based self-assembly approach that incorporates a diblock copolymer bearing electron- and ion-conducting blocks, poly(3-hexylthiophene)-*block*-poly(ethyleneoxide) (P3HT-*b*-PEO), with V_2_O_5_ to form a flexible, tough, carbon-free hybrid battery cathode. V_2_O_5_ is a promising lithium intercalation material, but it remains limited by its poor conductivity and mechanical properties. Our approach leads to a unique electrode structure consisting of interlocking V_2_O_5_ layers glued together with micellar aggregates of P3HT-*b*-PEO, which results in robust mechanical properties, far exceeding the those obtained from conventional fluoropolymer binders. Only 5 wt % polymer is required to triple the flexibility of V_2_O_5_, and electrodes comprised of 10 wt % polymer have unusually high toughness (293 kJ/m^3^) and specific energy (530 Wh/kg), both higher than reduced graphene oxide paper electrodes. Furthermore, addition of P3HT-*b*-PEO enhances lithium-ion diffusion, eliminates cracking during cycling, and boosts cyclability relative to V_2_O_5_ alone. These results highlight the importance of tradeoffs between mechanical and electrochemical performance, where polymer content can be used to tune both aspects.

There is a growing need for low-cost, flexible, and rugged energy storage devices compatible with emerging flexible energy conversion devices and structural energy and power^1^,^2^,^3^,^4^. Recent work has demonstrated electrode materials with both mechanical robustness and electrochemical functionality, including carbon nanotube Buckypaper^5^,^6^,^7^,^8^, reduced graphene oxide paper^9^,^10^,^11^, graphite on super-aligned CNTs^12^, TiO_2_ on activated carbon fabric^12^, and vanadium pentoxide (V_2_O_5_) wires^13^. Of these, V_2_O_5_ is particularly promising because it offers higher specific energy and because it has the ability to form paper-like electrodes very similar to Bucky paper. Unfortunately, the application of V_2_O_5_ in practical batteries has been hindered by a low Li^+^-diffusion coefficient (10^−12^ –10^−13^ cm^2^/s)^14^, low electronic conductivity (10^−2^ –10^−3^ S/cm)^14^ and volumetric changes^15^ during cycling.

Past approaches sought to address the electrochemical limitations of V_2_O_5_ by incorporation of conductive polymers, but little has been achieved by way of mechanical enhancement. Conjugated polymers can improve the electronic conductivity of V_2_O_5_ electrodes^16^,^17^, and ethylene oxide-containing polymers have demonstrated improved ionic conductivity^18^. Besides the materials themselves, the manner in which the blend is prepared can have a huge impact on electrode structure and mechanical properties. Large-scale phase separation between different components can result in poor mechanical properties, so intimate mixing and good interfacial adhesion is important.

To demonstrate a viable route to mechanically robust V_2_O_5_ electrodes, we propose the implementation of block copolymers, in which two polymers are covalently attached to each other preventing mascroscopic phase separation^19^. There exist only a handful of studies that incorporate block copolymers into hybrid electrodes, highlighting that the largest challenge for these hybrid electrodes is to balance the benefits of the added conductivity from the polymer without diluting the active material. For instance, the Balsara group reported the use of poly(3-hexylthiophene)-*block*-poly(ethyleneoxide) P3HT-*b*-PEO block copolymer as a conductive binder for a LiFePO_4_ cathode^20^,^21^. LiFePO_4_ performed at near-theoretical capacity, but a block copolymer loading of 50 wt% was required; mechanical properties were not reported. The Mayes group reported the synthesis of V_2_O_5_ aerogel within a block copolymer matrix^18^. This resulted in significantly improved mechanical properties, but the V_2_O_5_ content was only 34 wt%, resulting in a very low capacity. In both of these approaches, the loading of the active material was prohibitively low.

Here, our approach centers on a V_2_O_5_ hybrid electrode, in which we balance a high active material content with the benefits afforded by the P3HT-*b*-PEO block copolymer. The electrodes are prepared in a straightforward and scalable water-based process, and the addition of just 5 wt % block copolymer produces electrodes with excellent mechanical flexibility. We present the electrochemical and mechanical properties of the hybrid electrodes as a function of composition, for which a trade-off between polymer content and electromechanical properties is revealed. Specifically, we demonstrate that small amounts of a (P3HT-*b*-PEO) block copolymer can bring about significant improvements in mechanical flexibility, toughness, and lithium ion diffusion. The enhanced mechanical properties are attributed to the self-assembled layering of V_2_O_5_ with P3HT-*b*-PEO micellar aggregates. A proof-of-concept flexible half-cell is also shown. These results confirm that these ion- and electron-conducting block copolymers are excellent binders, bringing about not only conductivity but also mechanical flexibility.

## Results and Discussion

Our approach to flexible hybrid battery electrodes was to combine P3HT*-b*-PEO block copolymer with V_2_O_5_. The block copolymer contains electron- and ion-conducting P3HT and PEO blocks, respectively, whereas V_2_O_5_ has a high capacity for lithium ions. The challenge is to combine the two materials in such a manner so as to leverage the conductivity and flexibility of the polymer without compromising the electroactivity of the V_2_O_5_. Thus processing and blending were key considerations.

P3HT-*b*-PEO was synthesized using a modified approach based upon our prior publication^22^. Briefly, reactive, end-functionalized P3HT and PEO macroreagents were synthesized separately and coupled through a copper-catalyzed azide-alkyne click coupling reaction (see Scheme S1). The P3HT macroreagent was prepared through an externally-initiated polymerization reaction, which provided a higher degree of end-group functionality compared with conventional methods^23^. The catalyst contained a tosylate functionality, which was then converted to an azide in a single post-polymerization reaction step. The PEO-alkyne macroreagent was prepared through Steglich esterification of commercially available monomethoxy-poly(ethylene oxide) and hexynoic acid. The P3HT prepared for this study had a *M*_*n*_ of 13.3 kDa, as measured by NMR end-group analysis and multi-angle laser light scattering. The PEO block had a *M*_*n*_ of 7.1 kDa. The polydispersity of the final block copolymer was 1.24, as measured by gel-permeation chromatography (GPC). Full details on our synthetic procedure including ^1^H NMR characterization data and GPC data are provided in the Supporting Information, Figure S1.

We next desired to combine P3HT-*b*-PEO and V_2_O_5_ so as to obtain an intimately mixed, flexible, and electroactive electrode. Inspired by earlier approaches^16^,^18^,^24^,^25^,^26^,^27^, we first attempted *in situ* synthesis of V_2_O_5_ in the block copolymer as well as direct mixing of the two components in various solvents or as dry powders. However, none of these approaches yielded electrodes with appreciable electrochemical activity. This result might be explained by considering prior reports on polyaniline/V_2_O_5_ hybrid electrodes, where it was observed that polyaniline reduced V^5+^ to V^4+^ and the overall electrochemical activity was reduced^17^. In comparison, it is possible that similar interactions between P3HT and V_2_O_5_ might have led to the observed negligible electrochemical activity.

**Figure 1:**
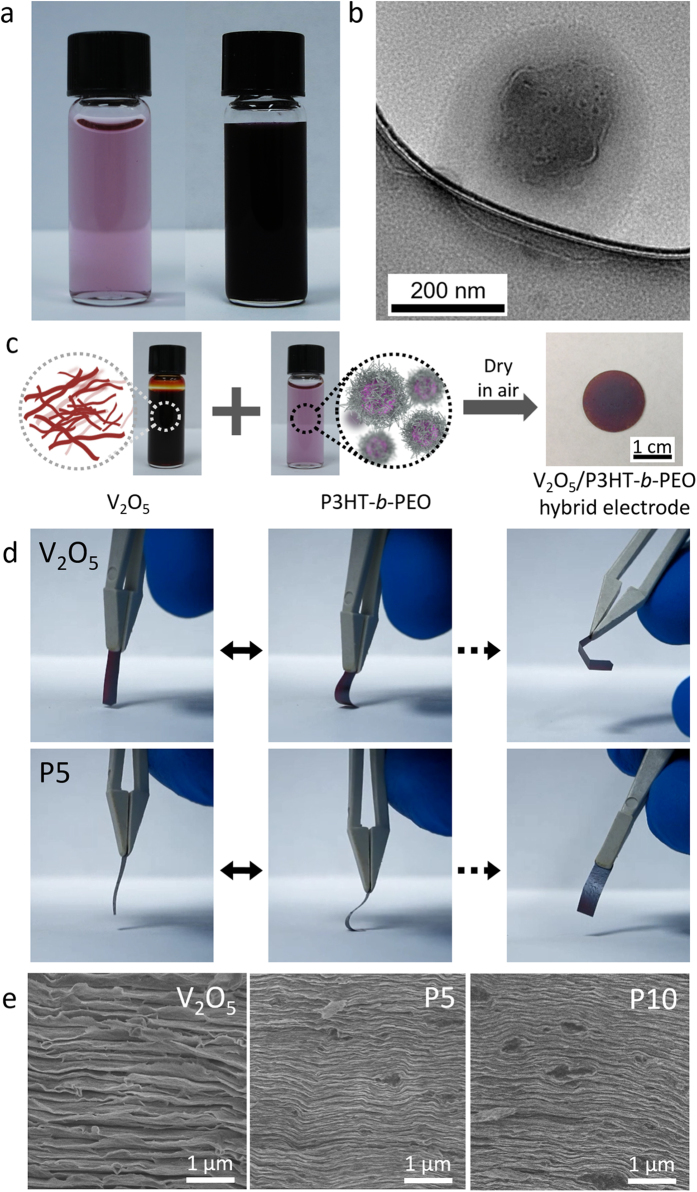
(**a**) P3HT-*b*-PEO dispersions at low concentration (0.05 mg/ml, left) and high concentration (1 mg/ml, right) with LiTFSI (the molar ratio of Li^+^ to PEO repeat units was 0.085) in water, (**b**) transmission electron micrograph of a drop-cast P3HT-*b*-PEO micellar aggregate. (**c**) Schematic of P3HT-*b*-PEO/V_2_O_5_/LiTFSI cathode preparation (drawn by H.A.). (**d**) Digital images of a V_2_O_5_/LiTFSI cathode (V_2_O_5_) (24 μm thick) and P5 (36 μm thick) in flexure. Movies are also provided in the Supporting Information. (**e**) Cross-sectional SEM images of V_2_O_5_, P5, and P10 cathodes after failure during dynamic mechanical analysis. Micellar aggregates (black dots) were arranged between V_2_O_5_ layers in hybrid electrodes.

An alternative water-based approach met with much greater success. First, P3HT-*b*-PEO and lithium bis(trifluoromethanesulfonyl)imide (LiTFSI) were dispersed in water using sonication. The molar ratio of Li^+^ to ethylene oxide repeat units was kept at 0.085^28^, which has been reported to be an optimal ratio for lithium-ion conduction^29^. Although P3HT-*b*-PEO is insoluble in water, after 1 h sonication, a uniform dispersion was formed (Fig. 1a). The block copolymer dispersion was stable for up to a week, after which large aggregates precipitated. The dispersion itself was purple in color and transparent. Other groups have used mixed solvents or dialysis to disperse P3HT-*b*-PEO block copolymers in water^30^,^31^,^32^, but our approach centers upon only sonication. From heareafter, hybrid electrodes containing *x* wt% polymer are called “Px”.

We propose that a micellization and aggregation process, in which hydrophilic PEO forms a corona around hydrophobic P3HT, is responsible for the dispersion’s stability. In support of this, TEM images of the drop-cast dispersion revealed a micellar aggregate diameter of ~320 nm, Fig. 1b. The dark core (~200 nm diameter) is likely the hydrophobic P3HT block, and the lighter shell (~60 nm in thickness) is likely the hydrophilic PEO block. The exact structure of the micellar aggregate remains a topic of future study. Dynamic light scattering (DLS) of the dispersion yielded an average aggregate diameter 250 nm (Figure S2), consistent with TEM results.

Having successfully made the P3HT-*b*-PEO dispersion, it was next mixed with aqueous V_2_O_5_ xerogel and sonicated, Fig. 1c. The mixture was then drop-cast, air-dried, and annealed at 90 °C. We prepared cathodes with total polymer content ranging from 1–50 wt % to assess the effect of block copolymer content on mechanical and electrochemical properties. Whereas neat V_2_O_5_ cathodes were brittle and failed under modest flexure, all hybrid cathodes were found to be flexible, (Fig. 1d, S3, Movies S1–S3). Cross-sectional SEM images reveal the fractured edges of neat V_2_O_5_, P5, and P10 cathodes. Well-packed V_2_O_5_ layers were visible throughout, Fig. 1e. The dark regions observed for P5 and P10 hybrid electrodes are likely micellar aggregates sandwiched between layers or locations at which aggregates had been pulled out during the fracturing process. SEM of the surface reveals a rough but uniform morphology, indicating that there is no large-scale phase separation (Figure S4). AFM images of the V_2_O_5_ and P5 cathode surfaces showed some evidence of micellar aggregates on the P5 electrode’s surface, Figure S5.

The microstructure of the P3HT-*b*-PEO/LiTFSI/V_2_O_5_ cathodes was analyzed using grazing-incidence wide-angle X-ray scattering (GIWAXS). Films for GIWAXS analysis were prepared by drop-casting onto glass slides followed by air-drying and vacuum annealing at 70 °C, similar to the procedure used for cathode preparation. As shown in Figure S6, P3HT-*b*-PEO/LiTFSI/V_2_O_5_ films exhibited wide-angle scattering peaks at *q* = 0.38 Å^−1^ and 0.64 Å^−1^, corresponding to primary scattering peaks for regioregular P3HT^33^ and V_2_O_5_·*n*H_2_O xerogels^5^, respectively. The most intense peak at 0.64 Å^−1^ corresponds to quasi-ordered V_2_O_5_·*n*H_2_O^34^. An additional peak at 1.70 Å^−1^ was also apparent from linecut analysis along the *q*_*z*_ direction, corresponding to the (003) plane of V_2_O_5_·*n*H_2_O^5^. Peaks at 0.38, 0.8, and 1.2 Å^−1^ correspond to regioregular P3HT crystallization in the edge-on orientation. An in-plane peak at *q*_*y*_ = 1.66 Å^−1^ was evident by linecut analysis and corresponds to face-to-face π-π stacking of P3HT chains.

These measurements reveal crystallization of P3HT and V_2_O_5_·*n*H_2_O in the composite cathodes. Scattering peaks match those for pure P3HT and V_2_O_5_·*n*H_2_O, suggesting phase segregation and crystallization of each component within discrete domains. Additionally, crystallites for P3HT and V_2_O_5_·*n*H_2_O are oriented in the out-of-plane direction, as commonly observed for thermally annealed thin films^33^. Differential scaning calorimetry revealed only a P3HT crystallization peak (Figure S7), which suggests phase segregation and crystallization of P3HT blocks but not of PEO, consistent with the GIWAXS. This indicates that PEO crystallization was restricted since polymer chains were intercalated into V_2_O_5_ layers.

**Figure 2:**
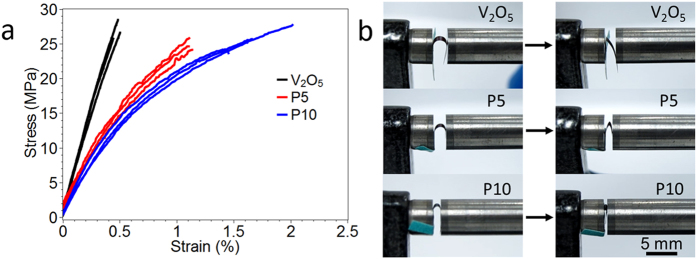
(**a**) Representative tensile profiles of hybrid electrodes in dependence of P3HT-*b*-PEO content and (**b**) digital images of collapsing radius test of hybrid electrodes. The collapsing radii of V_2_O_5_, P5, and P10 are 1.70, 0.40, and 0.30 mm, respectively. (Thickness of V_2_O_5_, P5, and P10: 26.9, 26.0, and 33.3 μm, respectively).

We next sought to quantify the mechanical properties using tensile testing and collapsing radius experiments. Figure 2a shows stress-strain curves for V_2_O_5_, P5, and P10 electrodes in triplicate, and Table 1 summarizes the average and standard deviation values of tensile strength, ultimate strain, Young’s modulus, and toughness (see also Table S1). Both V_2_O_5_ and hybrid electrodes exhibited tensile behavior very similar to that of graphene oxide paper, in which three regimes (straightening, elastic, and plastic) successively occur during elongation. P10 exhibited the highest ultimate strain and toughness, whereas the Young’s modulus decreased and the tensile strength remained unchanged. It is noteworthy that only 10 wt% polymer (P10) is required to obtain a 320% increase in toughness and a 250% increase in ultimate strain relative to polymer-free V_2_O_5_; this enhancement in mechanical properties does not come at the cost of tensile strength, but does cause a decrease in Young’s modulus. SEM images suggest that the enhanced ultimate strain and toughness enhancements come from well-dispersed P3HT-*b*-PEO micelles between V_2_O_5_ slabs, improving the flexibility of hybrid electrodes, Fig. 1e.

The mechanical properties of the hybrid electrodes are comparable, and in some cases higher, than that of graphene oxide paper and carbon nanotube sheets. For example the tensile strength of the P10 electrode was about four times higher than that of single-walled carbon nanotube Buckypaper (6.3 MPa), whereas the Young’s modulus was only slightly higher (3.5 vs. 2.3 GPa, respectively)^35^. In comparison, P10’s tensile strength and modulus was 66% and 16% that of graphene oxide paper of similar thickness (22–25 μm, average values of 39 MPa and 22 GPa, respectively). Notably, P10’s toughness was within range of that of graphene oxide paper (293 vs. 251 kJ/m^3^, respectively)^10^. On the other hand, the ultimate strain of P10 was far greater than that of graphene oxide paper (5.6% vs. 0.31%, respectively)^10^,^36^. These results show that the similarities in the mechanical properties between V_2_O_5_ hybrid electrodes, Buckypaper, and graphene oxide paper, which are largely attributed to the interlocking nature of the nanomaterials, similar to the morphology shown in Fig. 1e. A comparison with poly(vinylidene difluoride) (PVdF, Table 1) binder shows no remarkable improvement relative to P10, which suggests that the unique block copolymer structure is responsible for the enhanced mechanical properties.

Bending experiments were also conducted with hybrid electrodes, Fig. 2b, Table 1, and Table S2. A strip of a hybrid electrode with thickness *d*_*film*_ was bent so that a simple curve was formed and then compressed between two parallel plates, Figure S8. The radius of curvature *R*_*col*_ was taken at the first sign of kink formation. The normal strain *ε*_*x*_ at the surface was calculated by |*ε*_*x*_| = 0.5*d*_*film*_/*R*_*col*_^37^. The images in Fig. 2b clearly show that the samples sustained a greater collapsing radius as polymer content increased. This enhancement is attributed to the polymer, which acts as a glue, holding the V_2_O_5_ sheets together. Here too, PVdF binder showed no remarkable enhancement relative to P10. This result emphasizes that the P3HT-*b*-PEO polymer and the self-assembled structure is superior to conventional fluoropolymer binders in terms of mechanical performance.

We next turn to the electrochemical properties of hybrid electrodes. Both V_2_O_5_ and P3HT are capable of storing energy, but V_2_O_5_ is expected to dominate charge storage behavior because P3HT has a near-negligible capacity (less than 10 mAh/g)^20^,^21^. We found that for up to 50 wt% P3HT-*b*-PEO, the electrode showed no signs of dissolution into the electrolyte. However at higher polymer content, the electrolyte turned purple, suggestive of some amount of P3HT-*b*-PEO dissolution, Figure S9. Therefore, we investigated only hybrid electrodes containing up to 15 wt% polymer. At and above this concentration, the electrochemical performance was very poor, which we attribute to the low V_2_O_5_ content. A half-cell was assembled, and the hybrid electrode was used as the cathode, lithium foil as the anode, and 1 M LiTFSI in propylene carbonate as the electrolyte. Typical electrode thicknesses were in the range of 1.5 to 5 μm.

**Figure 3:**
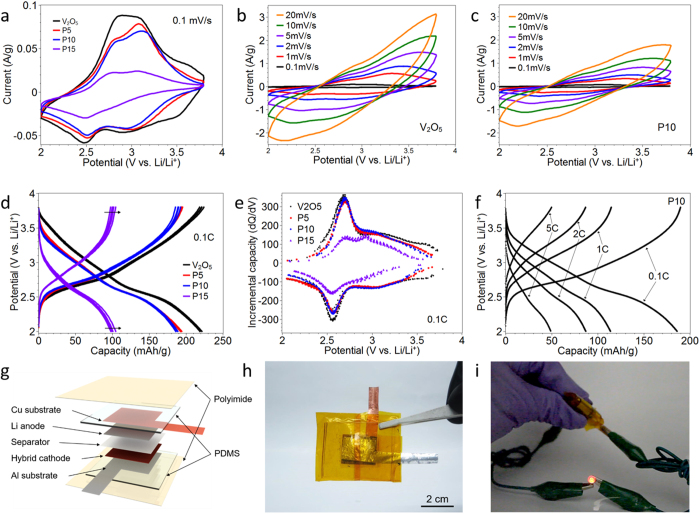
Cyclic voltammograms of (**a**) all compositions at 0.1 mV/sec, (**b**) V_2_O_5_ at various scan rates, and (**c**) P10 at various scan rates. All samples were approximately 4 μm in thickness. (**d**) Charge-discharge behavior of V_2_O_5_, P5, P10, and P15 electrodes at 0.1 C-rate and (**e**) incremental capacity (dQ/dV) taken from Fig. 3d. (**f**) Charge-discharge behavior at varying C-rates (0.1 to 5C) for a P10 cathode. For panels **a–f**, data were obtained from a two-electrode half-cell with lithium metal anode and 1 M LiTFSI in propylene carbonate. The capacity is based on mass of V_2_O_5_. (**g**) A schematic illustration of the assembled flexible half-cell using the P5 hybrid cathode and digital images of (**h**) the flexible battery as-assembled. (**i**) The flexible half-cell lights an LED while bending (also see Movie S4).

Cyclic voltammetry of the P3HT-*b*-PEO/V_2_O_5_|Li half-cell was carried out in the range of 2–3.8 V vs. Li/Li^+^ at a scan rate 0.1 mV/s for various polymer loadings, Fig. 3a. For V_2_O_5_, two distinct redox peaks appear at around 3.0 and 2.5 V in the cathodic/anodic scans consistent with insertions at a- and b-sites^26^,^38^,^39^. For compositions above 10 wt% P3HT-*b*-PEO, the current decreased significantly, suggesting diminished electrochemical activity. For V_2_O_5_ alone (Fig. 3b), as the scan rate increased, the V_2_O_5_ voltammogram became distorted, obscuring the higher voltage peak. On the other hand for P10 (Fig. 3c), little distortion was observed, suggestive of reduced Ohmic overpotential and increased accessibility of lithium ions into the xerogel^40^. The P3HT redox peak was observed only at very high polymer loadings; in Figure S10, P50 exhibited a small P3HT redox peak at 3.3 V^21^.

Figure 3d shows five galvanostatic charge-discharge cycles for P3HT-*b*-PEO/V_2_O_5_ hybrid electrodes of various compositions at a discharge rate of 0.1 C. From 3.8 to 2.0 V, a sloping discharge profile was obtained, typical of V_2_O_5_ xerogels^41^,^42^,^43^,^44^,^45^. The b-site Li^+^ intercalation reaction (at ~2.5 V) was also observed during cycling. V_2_O_5_ alone exhibited an average discharge capacity and Coulombic efficiency of 220.9 mAh g^−1^ and 99.4%, respectively. As the polymer content increased, the capacity decreased (P5: 193.3 mAh g^−1^ and P10: 187.2 mAh g^−1^) and the Coulombic efficiency decreased only slightly (P5: 99.5% and P10: 98.8%). The P5 electrode exhibited only a slight decrease in capacity relative to the pure V_2_O_5_ electrode, whereas the P15 exhibited a much larger decrease (100.2 mAh g^−1^). We also examined a thicker P10 electrode, where it was found that capacity dropped from 187.2 mAh g^−1^ to 40 mAh g^−1^ (5 um vs. 50 um, respectively), which we attribute to diffusion limitations in thicker electrodes. The derivative of the galvanostatic cycle was taken with respect to voltage to obtain the incremental capacity, Fig. 3e. The peaks displayed were similar to those observed in cyclic voltammograms. Cycling was also conducted at various C-rates, in which a drop in capacity with increasing C-rate was observed, Fig. 3f. From electrochemical impedance spectroscopy at 2.6 V vs. Li/Li^+^, where V_2_O_5_ is particularly active, the diffusion coefficients (*D*_Li_^+^) were calculated for V_2_O_5_, P5, and P10 cathodes^46^,^47^,^48^, Figure S11. The diffusion coefficient increased with polymer content, suggesting that lithium ion diffusion was enhanced by the P3HT-*b*-PEO diblock copolymer (*D*_Li_^+^ of V_2_O_5_, P5 and P10: 0.85 × 10^−11^, 1.64 × 10^−11^, and 2.09 × 10^−11^ cm^2^/s, respectively). A full study of the charge storage mechanism as a function of polymer content is underway.

To demonstrate the union of good mechanical properties with energy storage, we constructed a prototype flexible half-cell, Fig. 3h,i. The flexible battery was assembled with lithium ribbon as the anode and a P5 hybrid electrode as the cathode. Celgard film and 1 M LiTFSI in propylene carbonate were used as the separator film and electrolyte. Polydimethylsiloxane film with 1 mm thickness was used as packaging. To seal the cell more firmly, a polyimde film was used. The battery was repeated bent and flexed, and the LED light maintained its illumination, Fig. 3i and Movie S4.

**Figure 4:**
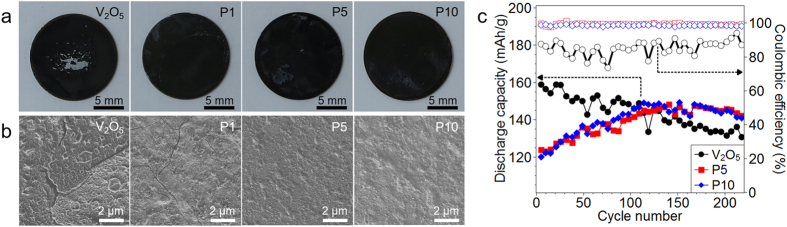
(**a**) Digital images of hybrid electrodes and (**b**) SEM images of hybrid electrodes after CV test. (**c**) Cycling behavior of V_2_O_5_, P5, and P10 electrodes at a discharge rate of 1C.

V_2_O_5_ is very sensitive to cycling, in which changes in volume have led to reduced cycle life and irreversible damage to the electrode. Post-mortem SEM analysis of our electrodes after cycling illustrate cracks, and digital images show large-scale flaking of the electrode, Fig. 4a,b and Figure S12. However with just 5 wt% polymer, the formation of cracks and flakes appears to be completely arrested. Accelerated cycling (Fig. 4c, Figure S13-S14) demonstrates a gradual capacity fade for V_2_O_5_, but P5 and P10 do not. Instead, the capacity of P5 and P10 gradually increases as electrolyte penetrates into the film with each cycle. After 100 cycles, the discharge capacity P5 and P10 electrodes exceeds that of V_2_O_5_. Furthermore, the Coulombic efficiency is enhanced for the P5 and P10 electrodes. These results demonstrate that the polymer acts as a good binder, and only a small amount is required to achieve good cyclability.

**Figure 5:**
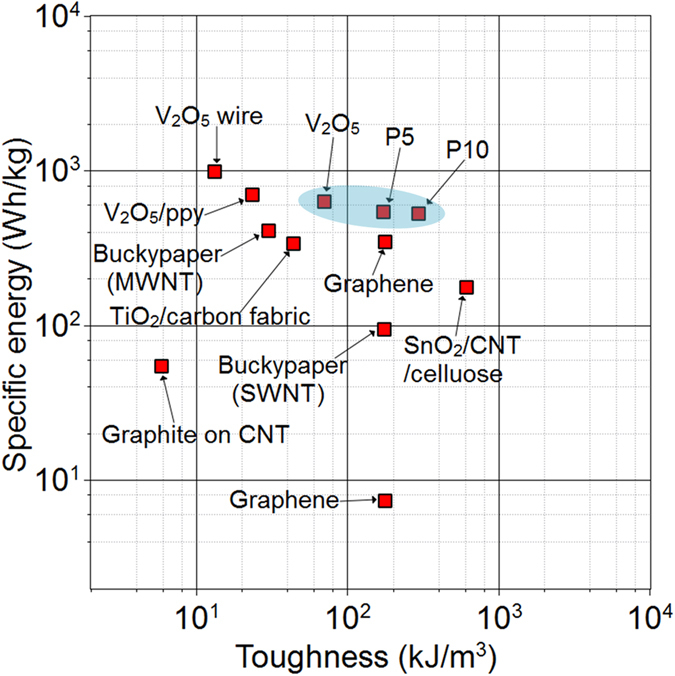
An Ashby plot of specific energy vs. toughness. Specific energy is reported as per mass of electrode. Data are taken from literature, discussed in main text, and data presented herein.

Figure 5 shows an Ashby plot of specific energy vs. toughness, summarizing data from the literature and data presented here. Since our focus in this study is the electrode, we present electromechanical properties for the electrode alone. Our electrodes show a good combination of both mechanical and electrochemical properties as compared to Buckypaper (SWNT^5^,^6^ and MWNT^7^,^8^), reduced graphene oxide paper^9^,^10^,^12^, graphite on super-aligned CNTs^12^, TiO_2_ on activated carbon fabric^12^, V_2_O_5_ wires^13^ and polypyrrole (ppy)-coated V_2_O_5_ wires^13^, Table S3. In the context of structural energy, it is important to note that one desires to maximize both mechanical and electrochemical properties. From the results, it is clear that it is very difficult to get both exceptional mechanical properties and electrochemical performance in one single electrode. One property often comes at the cost of another. One example of this here is with polymer content, in which increasing the polymer content enhances mechanical properties at the cost of capacity or energy. Still, for applications where both properties are desired it is more practical to find an electrode that marries the two even if there exists a trade-off.

## Conclusions

This paper outlines a simple, water-based route to self-assemble diblock copolymers bearing electron- and ion-conducting blocks with V_2_O_5_ to form a flexible hybrid battery cathode. Only a small amount of the diblock copolymer is required to realize significant gains in mechanical properties without significantly sacrificing electrochemical properties. Further, this polymer halted the progression of mechanical failure, a common problem for V_2_O_5_ electrodes, which suffer from severe volume expansion. In the context of structural energy and power, in which one must balance mechanical with electrochemical properties, these electrodes are particularly interesting. They are far more flexible than V_2_O_5_ alone, and they exhibit mechanical properties comparable with that of Bucky paper and reduced graphene oxide paper. Our future work will detail the morphology and structure of the electrode, along with a detailed analysis of charge storage mechanisms.

## Methods

### Materials

Vanadium pentoxide, lithium bis(trifluoromethanesulfonyl)imide (LiTFSI), poly(vinylidene fluoride) (PVdF, molecular weight = 534,000 g/mol), N-methyl-2-pyrrolidone (NMP), and propylene carbonate (PC) were purchased from Sigma Aldrich. Lithium ribbon was purchased from Alfa Aesar. All chemicals were used as received. 316 stainless steel coins used as substrates were purchased from MTI Corporation. Water was purified to 18.2 MΩ-cm (Milli-Q, Millipore). PEO-OH was purchased from from Aldrich (lot# BCBB1016, 7.17 kg mol^−1^ by ^1^H NMR).

### Synthesis of V_2_O_5_ xerogel and P3HT-*b*-PEO block copolymer

Described in the Supplementary Information.

### Preparation of polymer suspensions

A VWR Symphony ultrasonic cleaner (VWR Model 97043-936, 35 kHz) was used to prepare the polymer dispersion. P3HT-*b*-PEO suspensions were made by sonicating the polymer and LiTFSI in Milli-Q water at 0 °C to make a 4 mg/mL stock solution. The molar ratio of ethylene oxide units to lithium ions was 0.085. Before making composites, aliquots of 4 mg/mL P3HT-*b*-PEO solutions were diluted to 1 mg/mL. Hydrodynamic diameter was determined using dynamic light scattering (DLS) (ZetasizerNano ZS90, Malvern) at 25 °C. For DLS, the P3HT-*b*-PEO solution was diluted to 0.05 mg/ml.

### Cathode preparation

Before use, 316 stainless steel coins (12 mm diameter × 1.6 mm thickness) were cleansed *via* sonication for 15 minutes each in dichloromethane, acetone, water, and isopropanol, followed by drying under vacuum overnight at room temperature. Composites were made by mixing together appropriate volumes of 1 mg/mL P3HT-*b*-PEO (diluted from 4 mg/mL solutions) and 16.7 mg/mL V_2_O_5_ xerogel solutions in Milli-Q water. These solutions were drop-cast on stainless steel coins by depositing 0.97–1.12 mg worth of material (polymer + V_2_O_5_) onto the surface. Hereafter, electrodes containing X wt% polymers will be called “PX”. After drying in air, the cathodes were held under vacuum at 90 °C for 16 hours. For the electrodes containing PVdF, these were prepared by mixing V_2_O_5_ with PVdF in NMP. The mixture was cast as before and allowed to dry at 70 ^°^C for three hours, and then 90 ^°^C overnight under vacuum.

Cathode morphologies were investigated using scanning electron microscopy (SEM, JEOL JSM-7500F). Grazing incidence wide angle x-ray scattering measurements were carried out on Sector 8-ID-E at Advanced Photon Source, Argonne National Laboratory^49^. Beamline 8-ID-E operates at an energy of 7.35 keV and images were collected from a Pilatus 1MF camera (Dectris), with two exposures for different vertical position of the detector. After flatfield correction for detector nonuniformity, the images are combined to fill in the gaps for rows at the borders between modules, leaving dark only the columns of inactive pixels at the center. Using the GIXSGUI package^50^ for Matlab (Mathworks), data are corrected for x-ray polarization, detector sensitivity and geometrical solid-angle. The beam size is 200 μm (h) × 20 μm (v). Sample detector distance is 204 mm. Sample measurement and thermal annealing were carried out under vacuum which is in the range of 2 ~ 3 × 10^−6^ bar, with the sample stage interfaced with a Lakeshore 340 unit.

### Cell assembly and measurement

Electrochemical measurements were performed in two electrode cells (Tomcell Japan Co., Ltd.) assembled in a water-free, oxygen-free, argon-filled glovebox (MBraun) using lithium metal anodes. 1.0 M LiTFSI in propylene carbonate was used as the electrolyte and Celgard 3501 was used as the separator. Cyclic voltammetry, galvanostatic measurements, and impedance spectroscopy were performed using a Gamry Interface 1000, Solartron SI 1287, and Solartron 1470E.

### Free-standing film preparation and mechanical testing

P3HT-*b*-PEO/V_2_O_5_ mixtures were cast onto polystyrene weigh-boats (VWR), followed by air-drying. Following isolation from the weigh-boat, the hybrid electrodes were cut into rectangular strips of approximately 1 mm × 18 mm for testing. Static mechanical tensile tests were performed using a dynamic mechanical analyzer (DMA-Q800, TA Instruments). All tensile tests were conducted in controlled-force mode with a strain rate of 0.2%/min and preload of 0.02N.

## Additional Information

**How to cite this article**: An, H. *et al.* Highly Flexible Self-Assembled V_2_O_5_ Cathodes Enabled by Conducting Diblock Copolymers. *Sci. Rep.* **5**, 14166; doi: 10.1038/srep14166 (2015).

## Supplementary Material

Supplementary Information

Supplementary Movie S1

Supplementary Movie S2

Supplementary Movie S3

Supplementary Movie S4

## Figures and Tables

**Table 1:** Mechanical properties of hybrid electrodes derived from the stress-strain curves under tensile loading^a^ and from the collapsing radius test^b^ respectively.

Sample	Tensile strength^a^ σ (MPa)	Ultimate strain^a^ ε (m/m)	Normal strain^b^ |*ε*_*x*_| (m/m)	Young’s modulus^a^ E (GPa)	Toughness^a^ W (kJ/m^3^)
V_2_O_5_	27 ± 2	0.0048 ± 0.0004	0.008 ± 0.003	6.0 ± 0.7	70 ± 8
P5	25 ± 1	0.011 ± 0.002	0.034 ± 0.13	3.7 ± 0.9	171 ± 5
P10	26 ± 2	0.017 ± 0.003	0.056 ± 0.006	3.5 ± 0.3	293 ± 73
PVdF10^c^	24.1 ± 0.3	0.0078 ± 0.0006	0.010 ± 0.001	2.9 ± 0.6	99 ± 9

^a^Values obtained are an average of three samples for each tensile test.

^b^Values obtained are an average of three samples for each collapsing radius experiment.

^c^PVdF10 consists of 10 wt% PVdF and 90 wt% V_2_O_5_.
