# Supplementary material for: Highly Flexible Self-Assembled V2O5 Cathodes Enabled by Conducting Diblock Copolymers
Source: Sci Rep. 2015 Sep 22;5:14166. doi: 10.1038/srep14166 (PMC4585753; doi:10.1038/srep14166)
Supplement: Supplementary Information [file srep14166-s1.pdf]

Highly Flexible Self-Assembled V_2O_5 Cathodes Enabled by Conducting Diblock Copolymers

Hyosung An,[1] Jared Mike,[1] Kendall A. Smith,[2] Lisa Swank,[2] Yen-Hao Lin,[2] Stacy Pesek,[2] Rafael Verduzco,[2,3] and Jodie L. Lutkenhaus[1]**

1. Artie McFerrin Department of Chemical Engineering, Texas A&M University, College Station, TX

2. Department of Chemical and Biomolecular Engineering, Rice University, Houston, TX

3. Department of Materials Science and NanoEngineering, Rice University, Houston, TX

*jodie.lutkenhaus@che.tamu.edu, rafaelv@rice.edu

EXPERIMENTAL

Synthesis of V_2O_5 xerogel. Vanadium pentoxide xerogel was synthesized using hydrogen peroxide according to previously reported procedures.[1] Briefly, V_2O_5 (3.0 g) was dissolved into 300 mL of 10% aqueous H_2O_2 solution. This solution was aged for three days, after which the water was removed and the solid xerogel was dried in the oven under air overnight at 100°C. After synthesis, the V_2O_5 xerogel was resuspended in water at a concentration of 16.7 mg/mL.

Synthesis of P3HT-*b*-PEO block copolymer.

Isopropyl magnesium chloride with lithium chloride complex (^{i}PrMgCl·LiCl, 1.3M), tetrakis(triphenylphosphine)nickel(0) ($Ni(PPh_3)_4$), 4-chloro-3-methylphenol, 1,3-

bis(diphenylphosphino)propane (dppp), tetra-*n*-butylammonium fluoride (TBAF) (1.0 M in THF), 5-hexynoic acid (112.13 g/mol), 4-dimethylaminopyridine (DMAP) (122.17 g/mol), N,N,N′,N″,N″-pentamethyldiethylenetriamine (PMDETA) , 3-hexylthiophene, drisolve dichloromethane, p-Toluenesulfonyl chloride, azidotrimethylsilane, ethylene carbonate, imidazole, and copper (I) bromide, and *tert*-butyldimethylsilane were purchased from Sigma-Aldrich and used as received. 2,5-dibromo-3-hexylthiophene and 2-(4-chloro-3-methylphenoxy)ethanol[2] **(1)** were synthesized as previously reported. N-(3-dimethylaminopropyl)-N′-ethylcarbodiimide hydrochloride (EDC) was purchased from TCI America. All other solvents and reagents were purchased from VWR and used as received unless stated otherwise.

2-(4-chloro-3-methylphenoxy)ethyl tosylate (2)

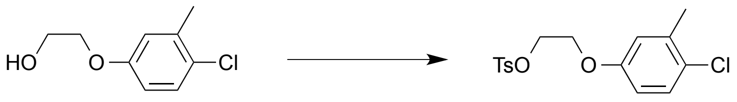

(**1**) (**2**)

A dry flask was charged with 2-(4-chloro-3-methylphenoxy)ethanol (**1**) (3.3 g, 17.7 mmol), p-Toluenesulfonyl chloride (6.8g, 35.7 mmol), dry dichloromethane (15ml), and pyridine (2.7 ml) and stirred overnight with a precipitate forming. The organic phase was dried over magnesium sulfate and purified by column chromatography (SiO_2, 25% EtOAc/hexanes). Solvent was removed under reduced pressure and the product was stored under vacuum overnight as a white solid. Yield (recovered): 3.47 g, 60%. ^{1}H NMR (500 MHz, $CDCl_3$), δ (ppm): 7.81 (d, 2H, Ar-*H*), 7.34 (d, 2H, Ar-*H*), 7.18 (d, 1H, Ar-*H*), 6.65 (d, 1H, Ar-*H*), 6.55 (q, 1H, Ar-*H*), 4.35 (t, 2H, Ar-O-CH_2-CH_2-O-), 4.10 (t, 2H, Ar-O-CH_2-CH_2-O-), 2.45 (s, 3H, Ar-CH_3), 2.31 (s, 3H, Ar-CH_3).

Tosylate-functionalized Ni(dppp) catalyst (3).

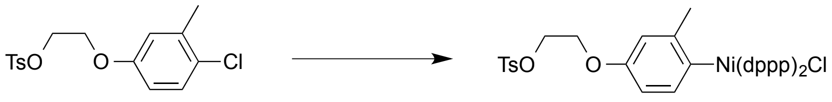

In a nitrogen-filled glovebox, $Ni(PPh_3)_4$ (54 mg, 0.049 mmol) was dissolved in 1 mL anhydrous tetrahydrofuran (THF). 2-(4-chloro-3-methylphenoxy)ethyl tosylate **(2)** (100 mg, 0.332 mmol) was added and the solution was allowed to stir overnight, at least 16 h. In a separate flask, 1,3-bis(diphenylphosphino)propane (80 mg, 0.192 mmol) was dissolved in 1 mL THF and added to the crude reaction solution of **(2)**, and the reaction mixture was stirred at room temperature for 2h. The crude product was removed from the glovebox and used to initiate the polymerization of P3HT, as described below.

Tosylate end-functionalized P3HT.

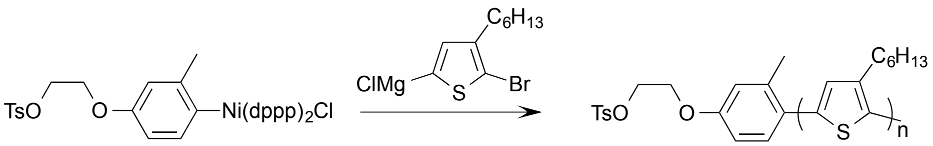

2,5-dibromo-3-hexylthiophene (1.91g, 5.86 mmol) was dissolved in anhydrous THF (4.5 mL) in a 100 mL round-bottom flask, and the solution was stirred at 0 °C for 15 minutes. A solution of isopropyl magnesium chloride with LiCl (1.3 M) in THF (4.51 mL, 5.86 mmol) was added, and the mixture was stirred for 2 hours at 0 °C. Next, 40 mL of THF was added to the reaction flask followed by the crude reaction solution containing (**3**) (0.049 mmol, 2mL). The solution was stirred for an hour and a half before quenching with 5M HCl (2 mL, 10 mmol). The polymer was recovered by precipitation in ethanol and dried under vacuum. ^{1}H NMR (500 MHz, $CDCl_3$), δ (ppm): 7.83 (d, 2H, Ar-*H*), 7.35 (d, 2H, Ar-*H*), 7.30 (d, 1H, Ar-*H*), 6.68 (d, 1H, Ar-*H*), 6.64 (q, 1H, Ar-*H*), 4.41 (t, 2H,

Ar-O-CH_2-CH$_2$-O-), 4.19 (t, 2H, Ar-O-CH$_2$-CH_2-O-), 2.45 (s, 3H, Ar-CH_3), 2.47 (s, 3H, Ar-CH_3), 7.00 (s, n·1H, Ar-*H*), 2.82 (t, n·2H,Ar-CH_2-), 1.72 (q, n·2H, -CH_2-), 1.46 (b, n·2H, -CH_2-), 1.37 (b, n·4H, -CH_2-), 0.93 (t, n·3H, -CH_3).

Azide end-functionalized P3HT.

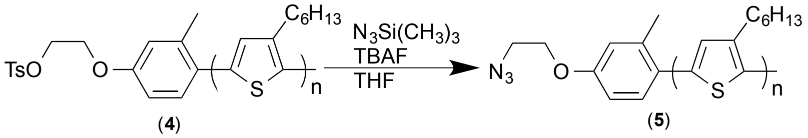

(**4**) (**5**)

In a typical procedure, tosylate functionalized P3HT (473 mg, 0.049 mmol) was dissolved in 20 ml of anhydrous THF and heated to dissolve the polymer before returning to room temperature. Azidotrimethylsilane (0.4 ml, 3mmol), 2ml of 1M TBAF (2mmol) were added to the flask and the solution was heated overnight at 35°C. Solution was precipitated in methanol, collected by filtration, and washed with acetone before drying overnight in a vacuum oven. Molecular weight and polydispersity was determined relative to polystyrene standards (M_N = 9.7 kg mol^{-1}, PDI = 1.22). Molecular weight was also determined by integration of aromatic hydrogens relative to end group hydrogens (M_N = 13.3 kg mol^{-1}). ^{1}H NMR (500 MHz, CDCl$_3$), δ (ppm): 7.38 (d, 1H, Ar-*H*), 6.84 (s, 1H, Ar-*H*), 6.80 (q, 1H, Ar-*H*), 4.20 (t, 2H, Ar-O-CH$_2$-CH_2-O-), 3.63 (t, 2H, Ar-O-CH_2-CH$_2$-O-), 7.00 (s, n·1H, Ar-*H*), 2.82 (t, n·2H,Ar-CH_2-), 1.72 (q, n·2H, -CH_2-), 1.46 (b, n·2H, -CH_2-), 1.37 (b, n·4H, -CH_2-), 0.93 (t, n·3H, -CH_3)

Synthesis of alkynyl-PEO.

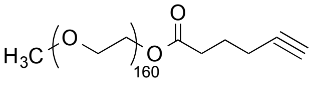

PEO-OH (5.07 g, 0.71 mmol), 5-hexynoic acid (1 g, 8.9 mmol), EDC (0.28 g, 1.466), DMAP (0.05 g, 0.42 mmol), and dichloromethane (30 ml) were added to a flask and

stirred overnight. Solution washed with brine and DI water in a separation funnel. Organic fraction collected, dried over magnesium sulfate, filtered and concentrated under reduced pressure. Cold ether was added (80ml) and the material collected by filtration and dried overnight under vacuum. Molecular weight and polydispersity was determined relative to polystyrene standards (M_N = 5.5 kg mol^{-1}, PDI = 1.06). Molecular weight was also determined by integration of aromatic hydrogens relative to end group hydrogens (M_N = 7.2 kg mol^{-1}). Yield 3.3 g (65%). ^{1}H NMR (500 MHz, $CDCl_3$), δ (ppm): 4.24 (t,2H,O-CH_2-CH_2-OCO), 3.64 (b, 652H, -O-CH_2-CH_2-O-), 3.78 (t,2H,O-CH_2-CH_2-OCO), 2.48 (t, 2H, OCO-CH_2-CH_2-CH_2-C-CH), 2.27 (t, 2H, OCO-CH_2-CH_2-CH_2-C-CH), 1.99 (s, 1H, C-CH), 1.85 (m, 2H, OCO-CH_2-CH_2-CH_2-C-CH)

P3HT-*b*-PEO block copolymers.

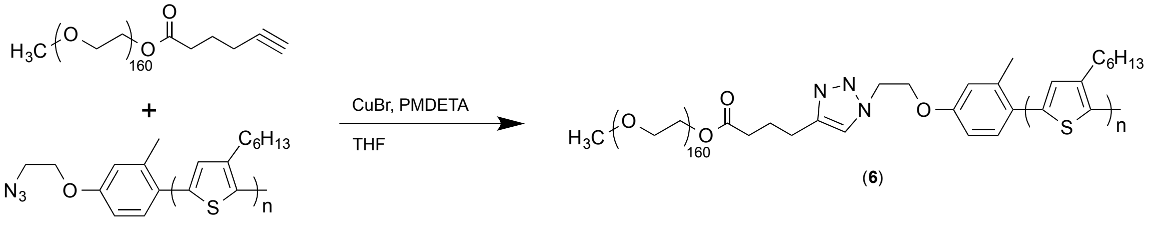

(**6**)

In a typical reaction, azide functionalized P3HT (403mg, 0.049 mmol), alkyne functionalized PEO (377 mg, 0.05 mmol), and CuBr (7.7mg, 54 mmol) where mixed with 20ml of THF in a dry flask. Oxygen was removed by bubbling with nitrogen for 20 min, after which PMDETA was added (0.2 ml). Reaction was allowed to proceed overnight at 40°C after which the raw product was passed through a short basic alumina column which was washed with copious amounts of THF, concentrated under reduced pressure, and washed with cold methanol to remove excess PEO. Sample is 76% P3HT by integration of backbone hydrogens from ^{1}H NMR. Molecular weight and polydispersity was determined relative to polystyrene standards (M_N = 9.35 kg mol^{-1}, PDI = 1.24). ^{1}H

NMR (500 MHz, $CDCl_3$), δ (ppm): 7.53 (s, 1H, CH triazole), 7.34 (d, 1H, Ar-*H*), 6.79 (s, 1H, Ar-*H*), 6.74 (m, 1H, Ar-*H*), 4.74 (t, 2H, Ar-O-$C\mathit{H}_2$-CH_2-O-), 4.37 (t, 2H, Ar-O-CH_2-$C\mathit{H}_2$-O-), 4.23 (t,2H,O-CH_2-$C\mathit{H}_2$-OCO), 3.78 (t,2H,O-$C\mathit{H}_2$-CH_2-OCO), 2.61 (t, 2H, OCO-$C\mathit{H}_2$-CH_2-CH_2-C-CH), 2.42 (t, 2H, OCO-CH_2-CH_2-$C\mathit{H}_2$-C-CH), 2.03 (m, 2H, OCO-CH_2-$C\mathit{H}_2$-CH_2-C-CH), 7.00 (s, n·1H, Ar-*H*), 2.82 (t, n·2H,Ar-$C\mathit{H}_2$-), 1.72 (q, n·2H, -$C\mathit{H}_2$-), 1.46 (b, n·2H, -$C\mathit{H}_2$-), 1.37 (b, n·4H, -$C\mathit{H}_2$-), 0.93 (t, n·3H, -$C\mathit{H}_3$) , 3.64 (b, m·H, -O-$C\mathit{H}_2$-$C\mathit{H}_2$-O-).

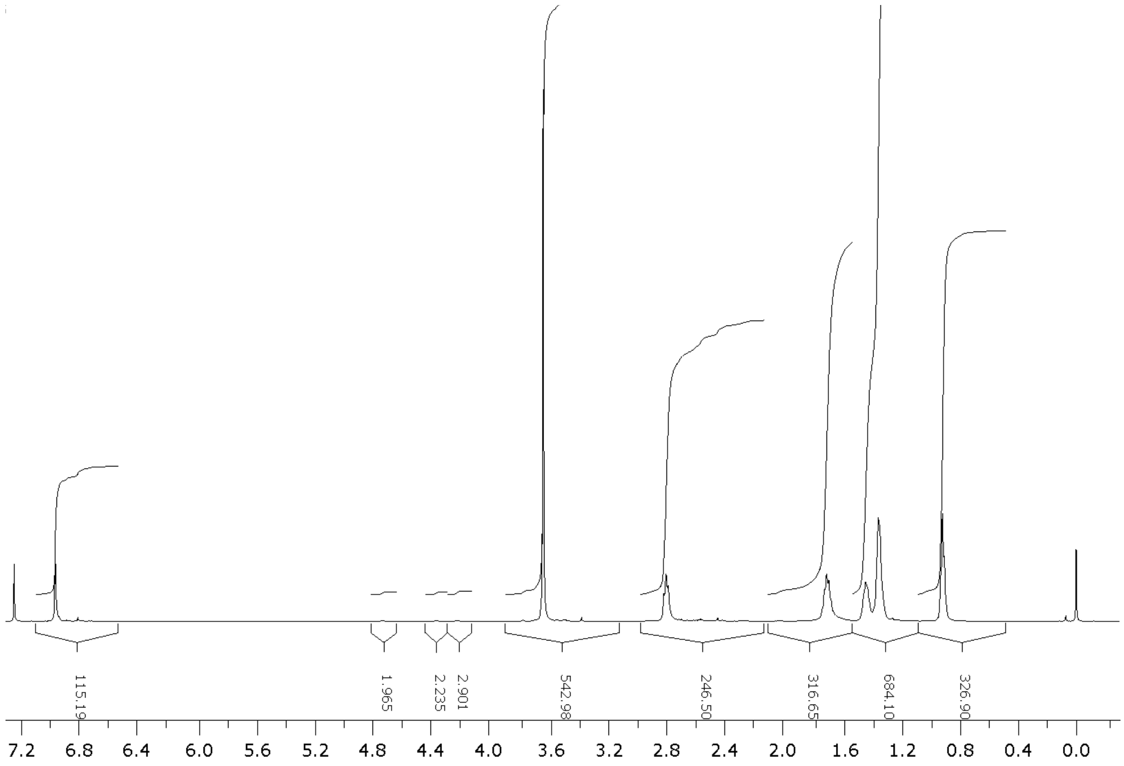

Figure S1. ^{1}H NMR of P3HT-*b*-PEO

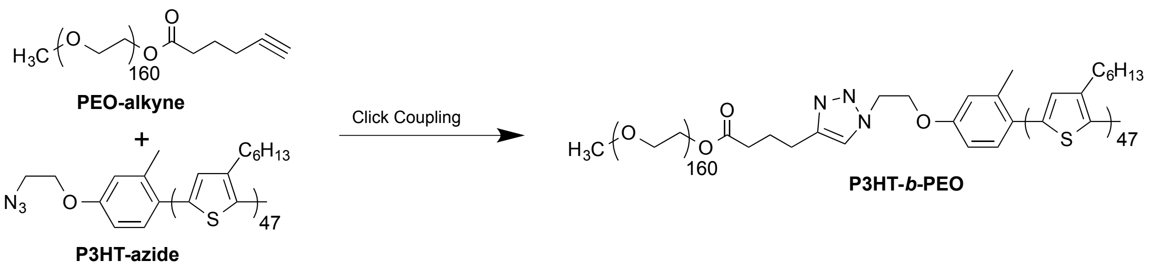

Scheme S1. Synthesis of P3HT-*b*-PEO block copolymers through click coupling. Degrees of polymerization shown for each polymer block are based on absolute M_n.

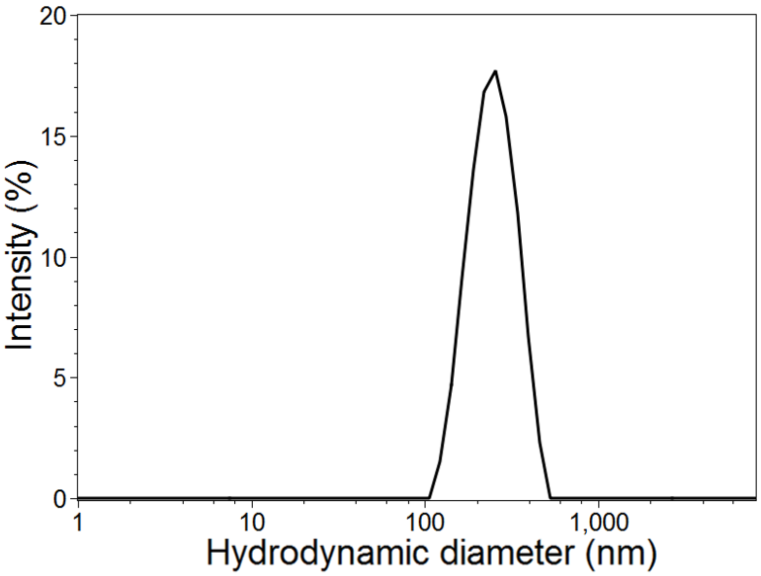

Figure S2. Dynamic light scattering of P3HT-*b*-PEO micelles in water.

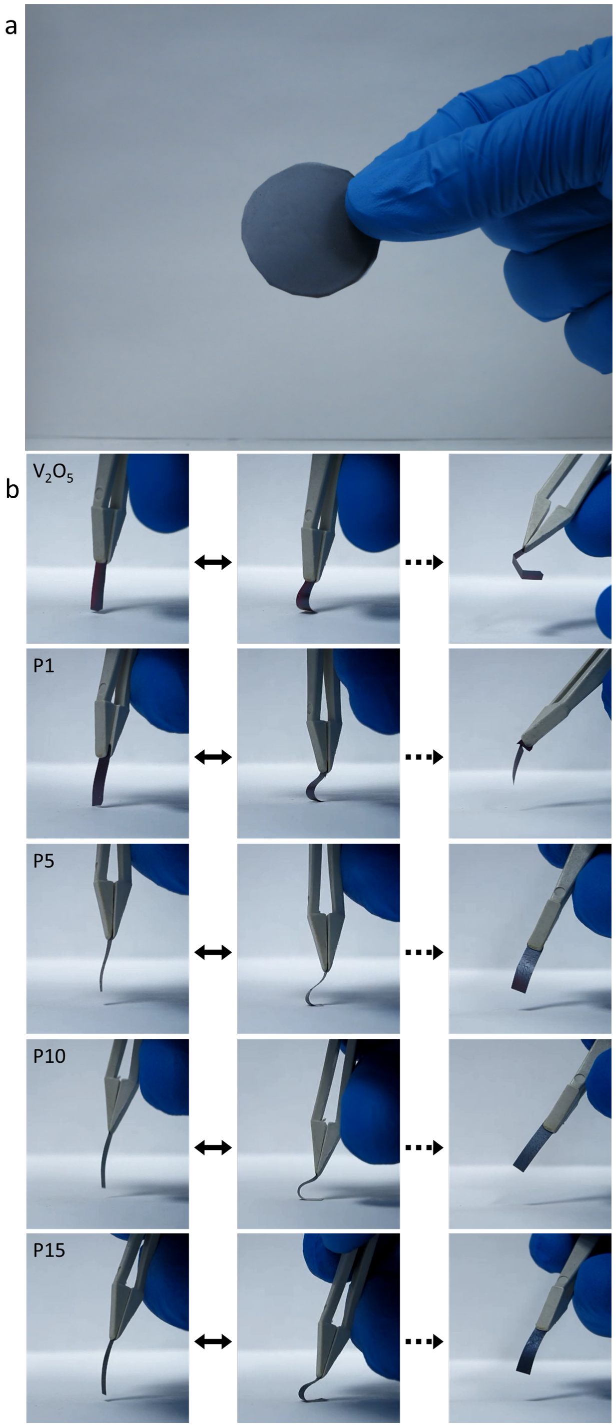

Figure S3. (a) A digital image of free-standing P5 electrode (3.3 cm diameter) and (b) images of free-standing hybrid electrodes demonstrating the flexibility of the films. Mechanical properties of hybrid electrodes improved with increasing P3HT-*b*-PEO content. A V_2O_5 film broke while bending several times. A P1 film broke after bending

dozens of times. P5, P10, and P15 could be flexed hundreds of times. Thickness of V_2O_5, P1, P5, P10, and P15: 24, 25, 36, 36, and 31 μm, respectively.

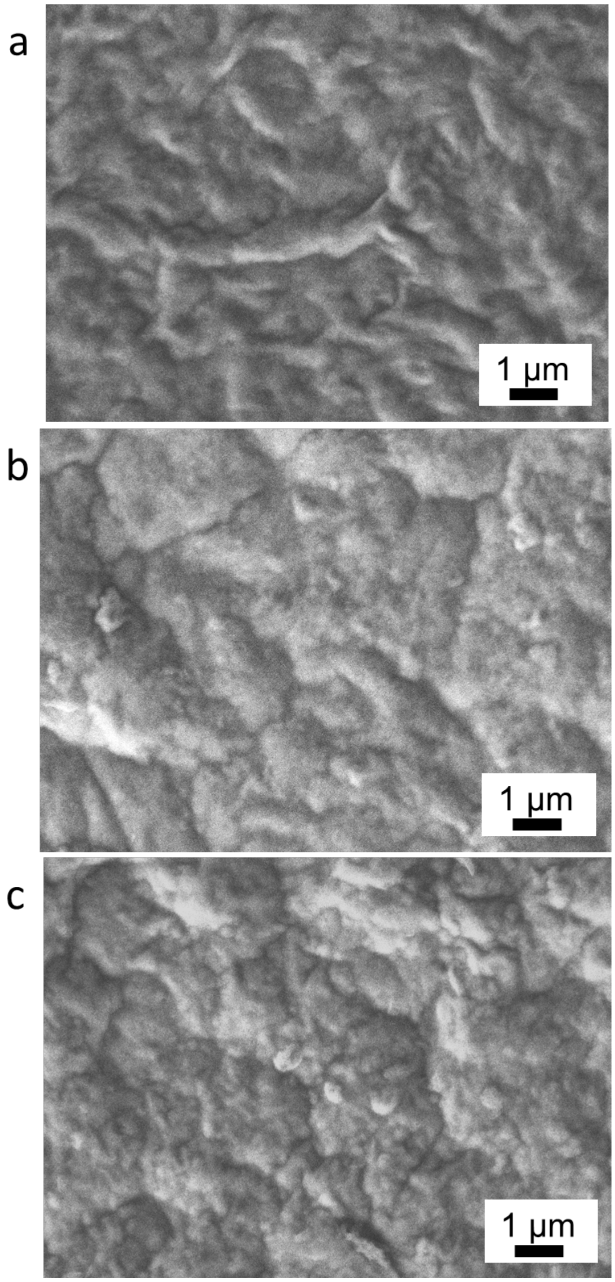

Figure S4. SEM images of (a) V_2O_5, (b) P5, and (c) P15 surfaces.

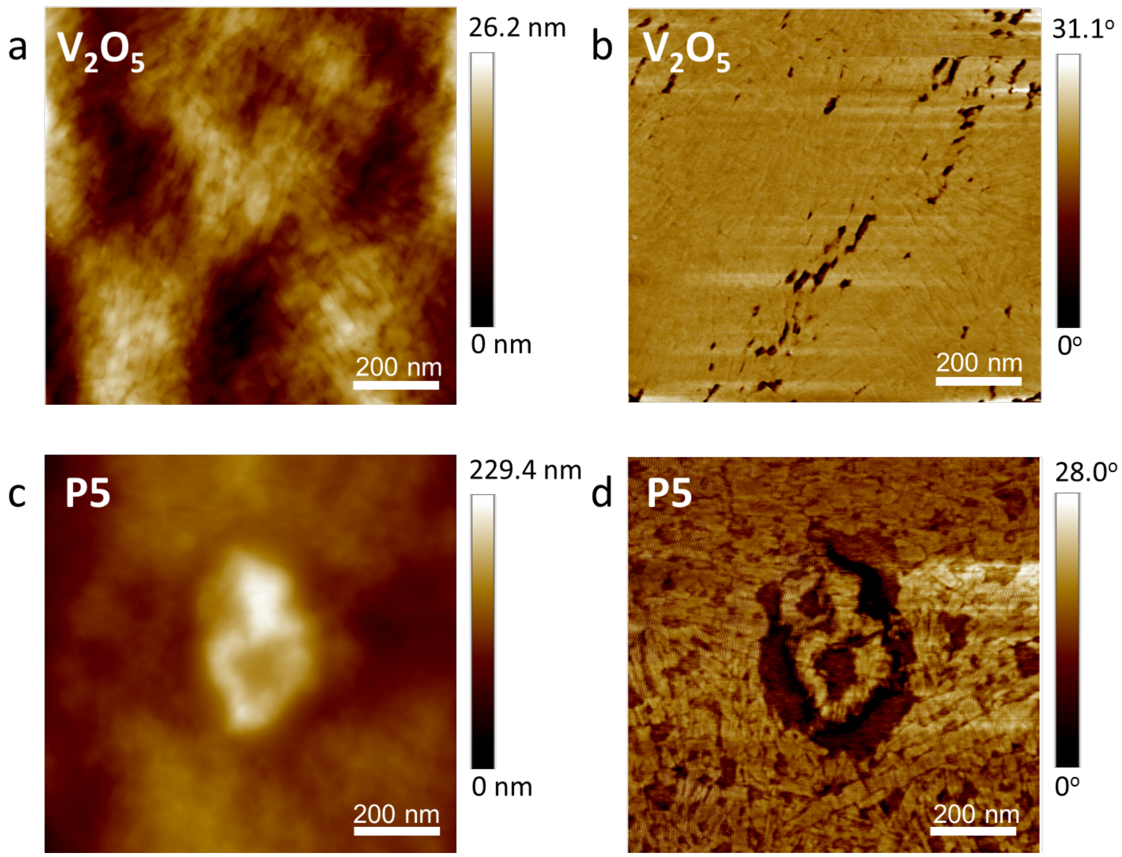

Figure S5. AFM images of (a-b) V_2O_5 xerogel and (c-d) of P3HT-*b*-PEO/V_2O_5/LiTFSI cathode with 5 wt% P3HT-*b*-PEO. Height (a,c) and phase (b,d) images are shown.

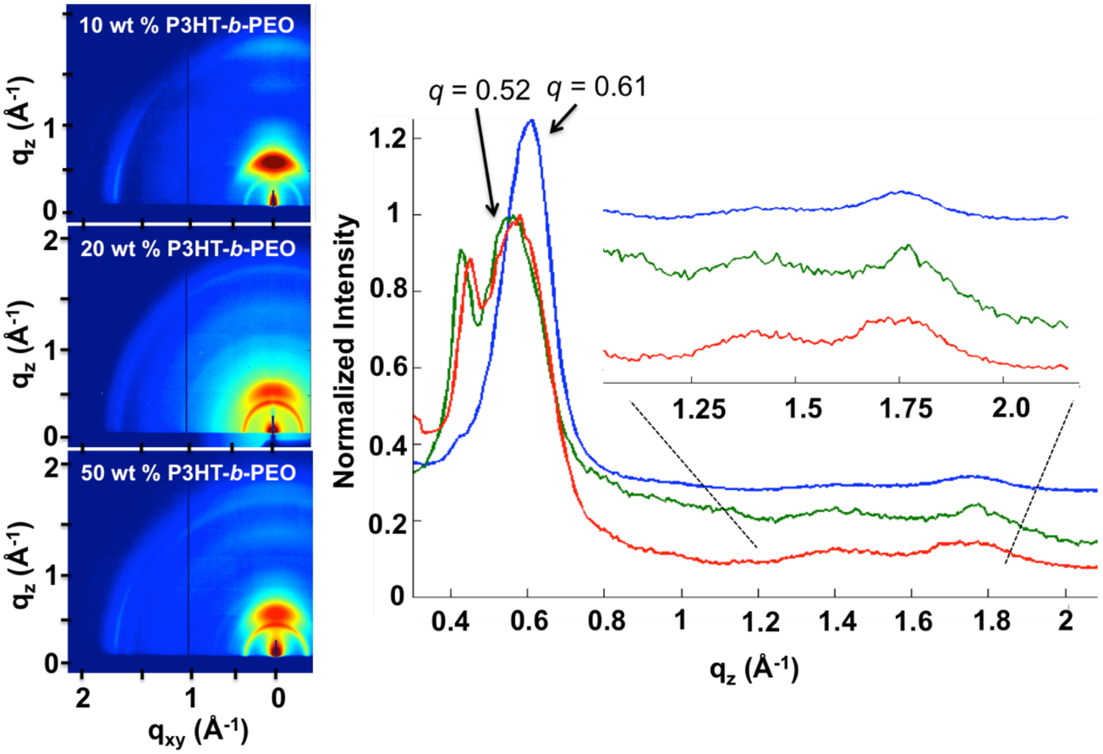

Figure S6. Grazing-incidence wide-angle X-ray scattering (GIWAXS) analysis of P3HT-*b*-PEO hybrid electrodes. 2-D GIXS scattering plots are shown on the left for P10, P20, and P50 hybrid cathodes. The plot on the right shows linecuts along the q_z direction, which corresponds to the direction perpendicular to the substrate.

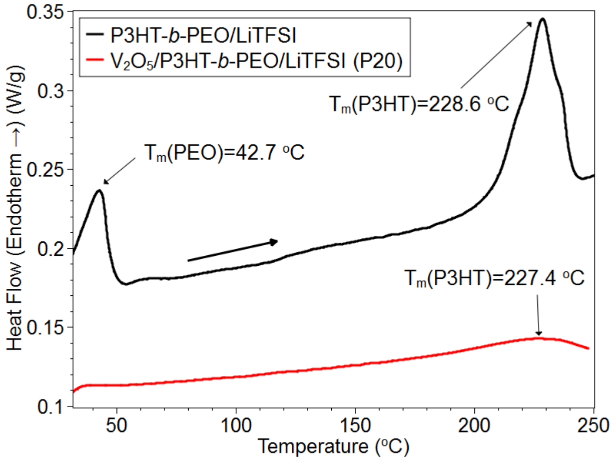

Figure S7. The second heating DSC scan of P3HT-*b*-PEO/LiTFSI and P20. (scan rate = 10 °C/min, 1.64 mg and 8.78 mg, respectively)

Table S1. Tensile testing results.

Sample_#	Length (mm)	Width (mm)	Thickness (µm)	Tensile strength σ (MPa)	Ultimate strain ε (m/m)	Young's modulus E (Gpa)	Toughness W (kJ/m^^{3})
V_2O_5_1	8.1792	0.726	24.1	25.72	0.0044	6.4	61.5
V_2O_5_2	7.7357	0.604	23.8	26.62	0.0050	5.2	73.2
V_2O_5_3	6.0937	0.77	34.0	28.46	0.0048	6.3	73.9
P5_1	10.911	0.888	14.3	25.81	0.0111	4.6	176.7
P5_2	5.892	0.804	11.2	24.17	0.0114	3.1	169.3
P5_3	9.5914	1.054	13.9	24.66	0.0110	3.4	168.1
P10_1	8.816	0.956	25.0	24.2	0.0146	3.8	235.7
P10_2	8.8212	0.848	29.6	27.73	0.0202	3.4	366.4
P10_3	9.195	0.918	28.5	25.7	0.0166	3.2	277.9

Tensile strength σ is stress at fracture. Ultimate strain ε is strain at fracture. Young's modulus E was determined by fitting the stress-strain curves ranging from initial point of

strain to 0.05%. Toughness W is the total work of extension to fracture, meaning total energy absorbed to fracture. Toughness was determined by taking the integral under the strain-stress curve.

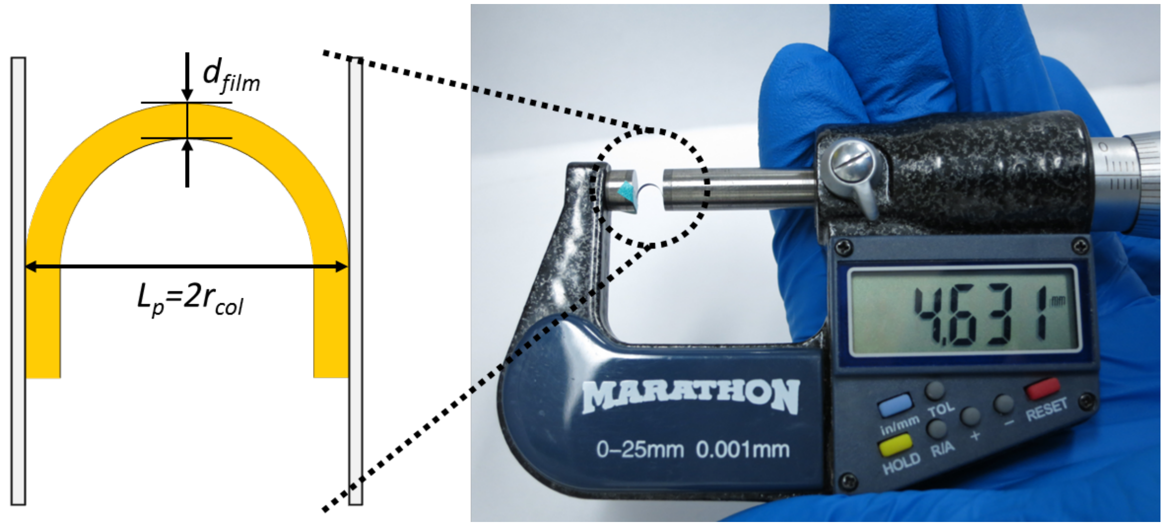

Figure S8. Digital image of collapsing radius test, and schematic diagram of thin film bent between plates.

Table S2. Mechanical properties of hybrid electrodes derived from the collapsing radius test

Sample	Collapsing radius R_{col} (mm)	Average thickness d_{film} (µm)	Normal strain $\|\varepsilon_x\|$ (m/m)
V_2O_5	1.70±0.85	26.9±9.1	0.008±0.003
P5	0.40±0.09	26.0±3.3	0.034±0.013
P10	0.30±0.04	33.3±8.3	0.056±0.006

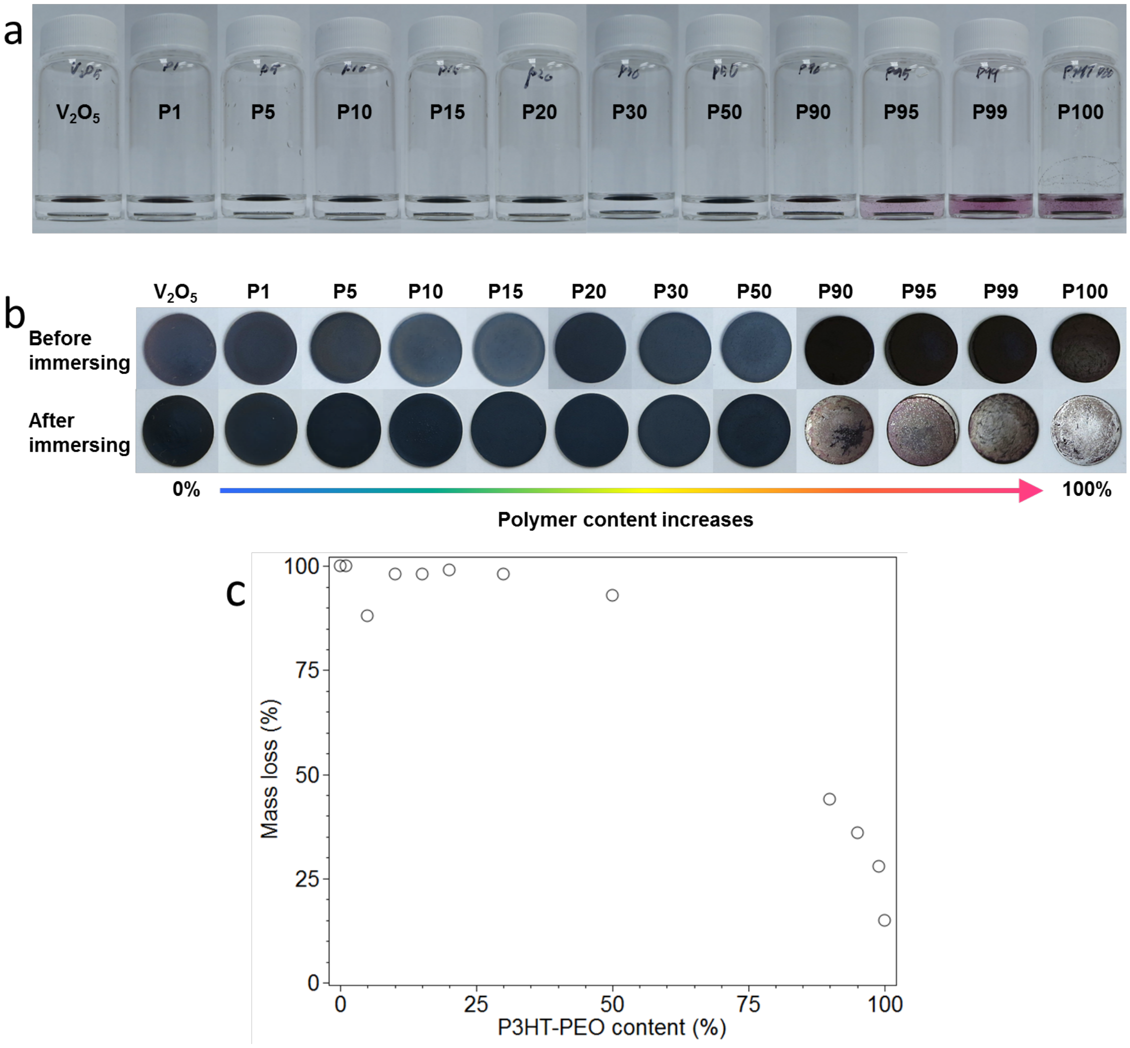

Figure S9. (a) Images showing solvent-resistance of hybrid electrodes with increasing P3HT-*b*-PEO concentration in propylene carbonate solvent after 1 week of soaking. (b) Hybrid electrodes immersed in propylene carbonate solution after 1 week. (From left, V_2O_5, P1, P5, P10, P15, P20, P30, P50, P90, P95, P99 and P100) (c) Plot of the mass loss of the hybrid electrodes in propylene carbonate as a function of P3HT-*b*-PEO concentration.

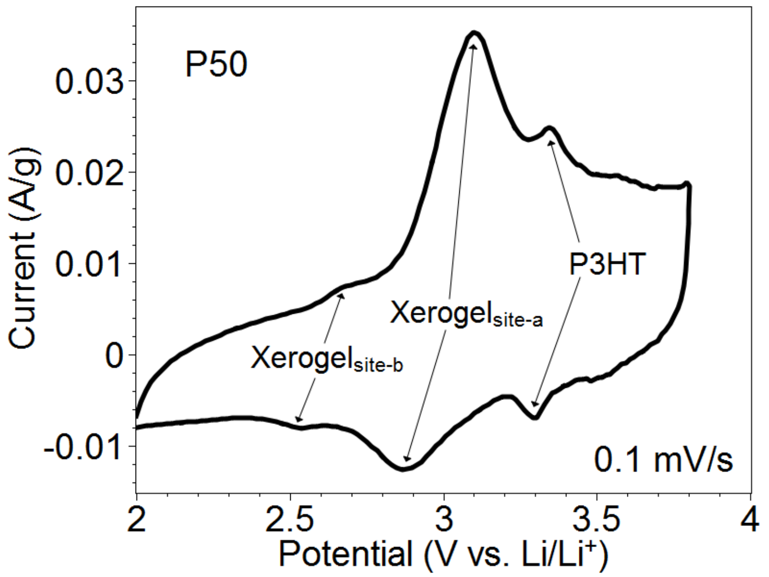

Figure S10. Cyclic voltammogram of P50 at 0.1mV/s. For the P50 hybrid electrode, a small P3HT redox peak was observed at 3.3V.[3] Due to its relatively small content in a cathode, P3HT redox peak was rarely observed in other hybrid electrodes containing less than 50 wt% of P3HT-*b*-PEO.

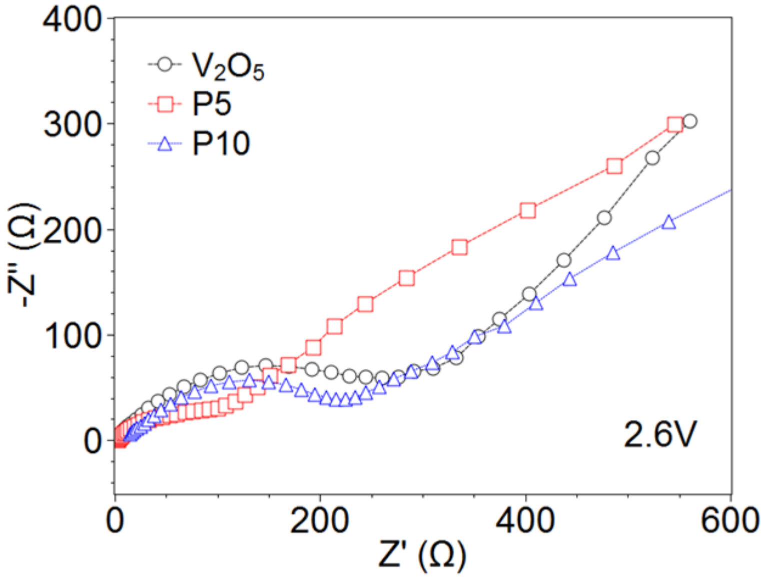

Figure S11. Electrochemical impedance spectroscopy (EIS) of V_2O_5, P5 and P10 at 2.6V. The amplitude was 10 mV. The frequency range was from 100,000 Hz to 0.05 Hz.

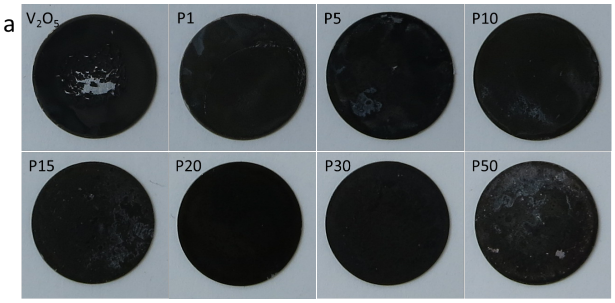

Figure S12. Digital images of hybrid electrode after electrochemical testing.

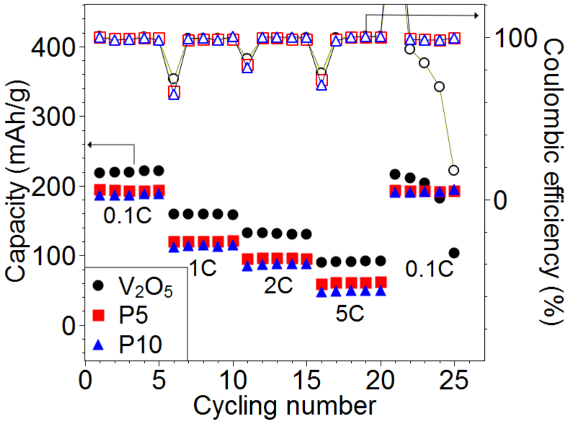

Figure S13. Half-cell capacities and Coulombic efficiencies under various C-rates for V_2O_5, P5, and P10.

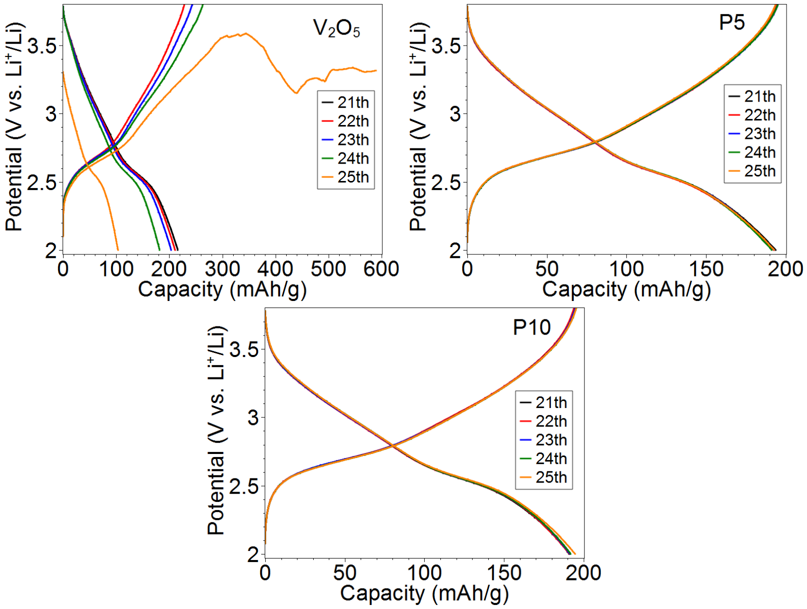

Figure S14. Charge-discharge profiles of V_2O_5, P5, and P10 during rate performance test.

It should also be noted that V_2O_5 samples had difficulty in reaching the upper voltage cutoff of 3.8V in the 25th charge step at 0.1 C-rate.

Table S3. Ashby plot data from Figure 5. Values are for the electrode alone.

Materials	Energy density (Wh/kg)	Tough-ness (kJ/m^3)	Fabrication method	
			Electrochemical performance	Mechanical performance
SWNT buckypaper[4,5]	95.0	174.0	Filtration of CNT with Triton X-100 as a surfactant[4]	Filtration of CNT with Triton X-100 as a surfactant [5]
MWNT buckypaper[6,7]	410.0	30.0	LbL method using MWNT-COOH/MWNT-NH_2 [6]	Electrophoretic deposition of MWNT with a surfactant [7]
Graphene[8,9]	7.4	177.5	Filtration of rGO[8]	Filtration of rGO with poly(sodium 4-styrenesulfonate) as a surfactant[9]
Graphene[9,10]	350.0	177.5	Filtration of rGO with CTAB as a surfactant[10]	Filtration of rGO with poly(sodium 4-styrenesulfonate) as a surfactant[9]
TiO_2 / activated carbon fabric (ACF)[11]	340.7	43.6	Hydrothermal reaction of TiO_2 onto ACF	
Graphite / CNT[12]	54.3	5.8	Casting mixture of Graphite/Super-P/PVDF (=8:1:1 wt. ratio) onto super-aligned carbon nanotube	
SnO_2 / CNT / carboxyl methyl cellulose (CMC)[13]	176.3	613.0	Casting mixture of SnO_2/CMC/MWCNT (= 3:1:1 wt. ratio) on Cu foil and then drying in the air followed by delamination	
V_2O_5 wire[14]	989.0	13.2	Filtration of V_2O_5 with Triton X-100 as a surfactant	

V_2O_5 wire/ polypropyrrole (ppy)[14]	704.0	23.2	Filtration of ppy-coated V_2O_5 with Triton X-100 as surfactant
V_2O_5 (this study)	631.1	69.5	Casting V_2O_5 xerogel
P5 (this study)	546.4	171.4	Casting mixture of V_2O_5/P3HT-*b*-PEO (=95:5 wt. ratio)
P10 (this study)	529.5	293.4	Casting mixture of V_2O_5/P3HT-*b*-PEO (=90:10 wt. ratio)

References

1 Park, N.-G. *et al.* Synthesis and electrochemical properties of V 2 O 5 intercalated with binary polymers. *Journal of power sources* **103**, 273-279 (2002).

2 Kempf, C. N., Smith, K. A., Pesek, S. L., Li, X. & Verduzco, R. Amphiphilic poly (alkylthiophene) block copolymers prepared via externally initiated GRIM and click coupling. *Polymer Chemistry* **4**, 2158-2163 (2013).

3 Patel, S. N., Javier, A. E. & Balsara, N. P. Electrochemically Oxidized Electronic and Ionic Conducting Nanostructured Block Copolymers for Lithium Battery Electrodes. *ACS Nano* **7**, 6056-6068, doi:10.1021/nn4018685 (2013).

4 Ng, S. *et al.* Single wall carbon nanotube paper as anode for lithium-ion battery. *Electrochimica Acta* **51**, 23-28 (2005).

5 Whitten, P. G., Spinks, G. M. & Wallace, G. G. Mechanical properties of carbon nanotube paper in ionic liquid and aqueous electrolytes. *Carbon* **43**, 1891-1896 (2005).

6 Lee, S. W. *et al.* High-power lithium batteries from functionalized carbon-nanotube electrodes. *Nature Nanotechnology* **5**, 531-537 (2010).

7 Rigueur, J. L., Hasan, S. A., Mahajan, S. V. & Dickerson, J. H. Buckypaper fabrication by liberation of electrophoretically deposited carbon nanotubes. *Carbon* **48**, 4090-4099 (2010).

8 Yang, X., Zhu, J., Qiu, L. & Li, D. Bioinspired Effective Prevention of Restacking in Multilayered Graphene Films: Towards the Next Generation of High‐Performance Supercapacitors. *Advanced Materials* **23**, 2833-2838 (2011).

9 Dikin, D. A. *et al.* Preparation and characterization of graphene oxide paper. *Nature* **448**, 457-460 (2007).

10 Ha, S. H., Jeong, Y. S. & Lee, Y. J. Free standing reduced graphene oxide film cathodes for lithium ion batteries. *ACS applied materials & interfaces* **5**, 12295-12303 (2013).

11 Liu, S. *et al.* A Flexible TiO2 (B)‐Based Battery Electrode with Superior Power Rate and Ultralong Cycle Life. *Advanced Materials* **25**, 3462-3467 (2013).

12 Wang, K. *et al.* Super‐Aligned Carbon Nanotube Films as Current Collectors for Lightweight and Flexible Lithium Ion Batteries. *Advanced Functional Materials* **23**, 846-853 (2013).

13 Klavetter, K. C. *et al.* A free-standing, flexible lithium-ion anode formed from an air-dried slurry cast of high tap density SnO 2, CMC polymer binder and Super-P Li. *Journal of Materials Chemistry A* **2**, 14459-14467 (2014).
14 Noerochim, L. *et al.* Impact of mechanical bending on the electrochemical performance of bendable lithium batteries with paper-like free-standing V 2 O 5–polypyrrole cathodes. *Journal of Materials Chemistry* **22**, 11159-11165 (2012).
